# Early transcriptional responses in *Solanum peruvianum* and *Solanum lycopersicum* account for different acclimation processes during water scarcity events

**DOI:** 10.1038/s41598-021-95622-2

**Published:** 2021-08-05

**Authors:** G. Tapia, M. González, J. Burgos, M. V. Vega, J. Méndez, L. Inostroza

**Affiliations:** 1 Unidad de Recursos Genéticos Vegetales, Instituto de Investigaciones Agropecuarias, INIA-Quilamapu, Avenida Vicente Mendez 515, Chillán, Chile; 2 Laboratorio de Microbiología Aplicada, Centro de Estudios Avanzados en Zonas Áridas (CEAZA), Raúl Bitrán 1305, La Serena, Chile

**Keywords:** Plant sciences, Plant domestication, Plant physiology, Plant stress responses

## Abstract

Cultivated tomato *Solanum lycopersicum* (*Slyc*) is sensitive to water shortages, while its wild relative *Solanum peruvianum* L. (*Sper*), an herbaceous perennial small shrub, can grow under water scarcity and soil salinity environments. Plastic *Sper* modifies the plant architecture when suffering from drought, which is mediated by the replacement of leaf organs, among other changes. The early events that trigger acclimation and improve these morphological traits are unknown. In this study, a physiological and transcriptomic approach was used to understand the processes that differentiate the response in *Slyc* and *Sper* in the context of acclimation to stress and future consequences for plant architecture. In this regard, moderate (MD) and severe drought (SD) were imposed, mediating PEG treatments. The results showed a reduction in water and osmotic potential during stress, which correlated with the upregulation of sugar and proline metabolism-related genes. Additionally, the senescence-related genes FTSH6 protease and asparagine synthase were highly induced in both species. However, GO categories such as “protein ubiquitination” or “endopeptidase inhibitor activity” were differentially enriched in *Sper* and *Slyc*, respectively. Genes related to polyamine biosynthesis were induced, while several cyclins and kinetin were downregulated in *Sper* under drought treatments. Repression of photosynthesis-related genes was correlated with a higher reduction in the electron transport rate in *Slyc* than in *Sper*. Additionally, transcription factors from the ERF, WRKY and NAC families were commonly induced in *Sper*. Although some similar responses were induced in both species under drought stress, many important changes were detected to be differentially induced. This suggests that different pathways dictate the strategies to address the early response to drought and the consequent episodes in the acclimation process in both tomato species.

## Introduction

Water availability for agriculture is decreasing in many places around the world, with bad irrigation practices, climate change and industrial uses being the major causes. At the same time, the requirements for food production are also increasing, which introduces a challenge to improve the efficiency of water use without reducing the yield of crops^1^. A strategy to improve crops is to use genetic resources. Wild species related to agricultural crops (wild relatives) possess the ability to grow under different environmental conditions, being a key reservoir to extend the genetic base of agricultural traits and to use new allelic variations to increase the adaptation of cultivated plants in both biotic and abiotic stress^2–4^.

The cultivated tomato *Solanum lycopersicum* is an economically important fruit that belongs to the monophyletic Solanaceae^5^, a group of species that are distributed worldwide in a wide range of environmental conditions, including tropical and dry climates^6^. While *Solanum lycopersicum* is reported to be sensitive to water shortages, some wild related Solanaceae species can inhabit extreme environmental conditions, such as *S. peruvianum, S. lycopersicoides, S. sitiens, S. pennellii* and *S. chilense*^7^.

Previously, we studied the effect of low water irrigation on the acclimation of *S. lycopersicum* (*Slyc*) and *S. peruvianum (Sper)* to water deficit. During the water treatments, the plants suffered successive cycles of drought stress and recovery before reaching an acclimation state. It promotes anatomical, morphological and physiological changes in leaf traits. The modifications mediated by water restriction play an important role in tolerance to drought stress in herbaceous plants^8^.

Few studies are available about the early response of plants in terms of signaling and physiological and molecular mechanisms related to drought tolerance, and fewer are available about differential responses between drought-tolerant and drought-sensitive species during acclimation. Studies conducted by Harb et al.^9^ described three states of acclimation in Arabidopsis from an early state characterized by stress perception, signaling and reprogramming of gene expression. An early response implies a combination of endogenous hormones, such as high JA and ABA levels, that induce an acclimation response^9^. Although several processes are activated rapidly in plants during short-term drought stress, such as stomatal closure, others, such as the accumulation of compatible osmolytes, an increase in sugar catabolism, induction of the ROS scavenging system and modification of cellular lipid composition, are also important. On the other hand, senescence has also been demonstrated to play an important role in drought resistance^10^. This process is induced in a second phase, being associated with ABA signaling, and apparently could help to generate a greater osmotic potential gradient, causing a preferential flow of water to the newly developed tissues. In addition, the senescence of old tissues also promotes acclimation and the development of new and more resistant tissues^11^.

Hormone signaling based on ABA, ethylene or jasmonates activates both common and different groups of genes under abiotic stresses^12–14^. Hormones trigger a signal transduction cascade that includes protein kinases, phosphatases and transcription factors (TFs), which synchronize the timing of gene expression during the stress response^15^. In this sense, different families of transcription factors have been described to be involved in the drought response^16–18^, which regulate several groups of genes related to amino acid and carbohydrate metabolism and cuticle biosynthesis or are associated with cell protection^12, 19, 20^. Although TFs can increase the drought tolerance of plants, they can also contribute negatively to other important processes, as their growth is necessary to accurately identify the mechanisms used by plant species adapted to adverse conditions^21^.

Currently, next-generation sequencing (NGS) has enabled the study of transcriptomes (RNA-seq). This technology describes differentially expressed genes with critical roles in stress adaptation in non-model species^22, 23^.

Considering that plant restructuration during acclimation to water deficit requires early changes in gene expression, we analyzed the transcriptome of two contrasting drought-tolerant tomato species in an early phase under two severity conditions. Enriched gene groups for both species showed several differences, which are discussed here. Water stress severity also induced differential gene expression between the treatments, which can be associated with subsequent morphological and physiological changes in the acclimated plants. Specific groups of genes from GO were analyzed in detail. All of the findings suggest the presence of contrasting mechanisms to face drought stress in both species during the early response. The implications of both responses are discussed as a function of the mechanism of drought tolerance.

## Materials and methods

### Plant material

Seeds of *Slyc* (var Money maker) and *Sper* (Acc. Q958) were provided by Germplasm Bank Network of the Instituto de Investigaciones Agropecuarias (INIA) under a standard material transfer agreement and an Institutional INIA Policy for ABS of plant genetic material following international agreement signed by Chile. *Solanum peruvianum* is at least concern according to IUCN Red List. https://www.iucnredlist.org/species/71783673/71783874.

Seeds were germinated in speedling with a vermiculite-turbe mixture (1:1). Seedlings were grown under optimal conditions (temperature = 24 °C, photoperiod = 16/8 h light/dark, light intensity = 250 μmol/m^2^). The plantlets were irrigated with a solution of phostrogen fertilizer (Compo, Germany) and grown for 6 weeks.

For long-term water stress treatment, plants were initially not irrigated until wilting symptoms appeared. During this period, RWC was recorded until reaching 50% in the leaves. RWC was measured as described by Pieczynski et al.^24^. The plants were then irrigated with 40 mL of water. When the plants were newly turgid, the first cycle of dehydration/rewatering (D/RW) was achieved. The timing of the successive water applications in the next cycles was determined by the use of a soil water sensor (GS1, Decagon Device Inc., Pullman, WA, USA) according to the equivalence of RWC.

To evaluate the response of tomato species to equivalent conditions, short-term water stress treatment was applied by soaking the trays with a solution of PEG 8000. For moderate (MD) and severe drought (SD), 5% and 12% PEG 8000 were used, respectively, for 24 h. Nonstressed plants were soaked with distilled water. Two sets of plants were separated for each treatment. The first set was used for RNA extraction from leaves, while the second set was used for the measurement of chlorophyll fluorescence, water and osmotic potential and proline.

### Physiological and biochemical measurement

The osmotic potential (Ψπ) was measured from 0.3 g of frozen leaf tissue, which was ground in a microtube. Aliquots of 10 µL were obtained for measurement in a VAPRO 5600 vapor pressure osmometer (Wescor, Logan, UT USA). The results obtained as mmol kg^−1^ were transformed to MPa using the van’t Hoff equation: MPa = [0.173 − (0.0269)(× mmol kg^−1^)]*0.1 described by Inostroza et al.^25^. Water potential was measured in the detached third leaf using a Scholander-type pressure chamber (3000 Series; Soil moisture Equipment, Santa Barbara, CA, USA).

The maximal efficiency of PSII was determined using a modulated fluorometer (FMS II, Hansatech Instrument, UK) and calculated as F_v_/F_m_ = F_0_ − F_v_/F_m_. The rate of electron transport was calculated as ETR = ΦPSII*PPDF *aβ, where PPDF is the photosynthetic photon flux density, “a” is the leaf absorbance and β is the distribution of energy absorbed between both photosystems (0.5). The leaf absorbance was measured using the IMAGING PAM chlorophyll fluorometer (Walz, Germany).

For proline determination, 0.2 g fresh sample was homogenized with liquid N in a mortar, adding 5 ml of 3% sulfosalicylic acid. The proline content was quantified through colorimetric determination with acidic ninhydrin reagent at 520 nm, according to Bates et al.^26^. The spectrophotometric determinations were performed using an EPOCH Microplate UV–Vis Spectrophotometer (Biotek Instruments, Winooski, VT, USA) with COSTAR 3596 (Corning, New York, NY, USA).

### RNA isolation, library construction and deep sequencing

Two biological replicates of 6 treatments (C-*Sper*, C-Slyc, MD-*Sper*, MD-*Slyc*, SD-*Sper* and SD-*Slyc*) summarizing 12 samples were obtained from a pool of six plant leaves. Each sample was ground in a mortar to a fine powder, and total RNA was isolated using the SV total RNA isolation system (Promega, Madison, WI, USA). Total RNA was quantified using an Epoch microvolume spectrophotometer system (Biotek). RNA integrity was evaluated by denaturing gel electrophoresis and a Bioanalyzer 2100 (Agilent Technologies, CA) with an RNA integrity number (RIN) greater than or equal to 8. Library construction and deep sequencing for each sample were performed in a contracted sequencing facility (Macrogen, Inc. Seoul, South Korea). The Illumina HiSeq2000 platform with the previous construction of a TruSeq mRNA library for paired-end application was selected. It was performed under paired ends for a read length of 100 bp, obtaining a total of approximately 10–14 million filtered reads for each library independently. The transcriptome data are available in NCBI Bioproject Code: PRJNA641399.

### RNAseq data processing, differential gene expression (DEG) analysis and functional classification of DEGs

The raw data were preprocessed using Prinseq software to remove the low-quality data, Illumina adapter sequences and readings of less than 50 bp^27^. In addition, three Q quality scores were assayed (Q20, Q25 and Q30), and Q = 30 was finally selected. Then, HISAT2 software (version: 2.2.1)^28^ was used to map clean reads to the tomato (*Solanum lycopersicum*) reference genome version ITAG 4.1. Afterwards, gene abundance estimation was performed using Stringtie software (version: 2.1.2), and the count read matrix values for known gene models were determined using the provided python script prepDE.py^28^. Count read values were analyzed using the EdgeR package from R software to identify the differentially expressed genes (DEGs)^29^. Finally, a significance threshold value was determined by FDR (false discovery rate) < 0.05 and fold-change ≥ 4.

Finally, to identify GO categories of differentially expressed genes, InterProScan software was used^30^. The results were classified into GO categories using GOATOOLS V.2.7 and ShinyGO V.0.61 software^31, 32^. Heatmap plots for DEGs were previously transformed to Log_2_(Count + 1) and visualized with the pHeatmap package using ward with the D2 clustering method.

### Gene expression analysis mediating qRT-PCR

Eight genes selected from RNA-seq were studied using qRT-PCR analysis, while ubiquitin (UBI), elongation factor (EF1a) and AP-2 complex subunit mu (CAC) were used as housekeeping genes. First strand cDNA was synthesized with a superscript RT-PCR system (Thermo Fisher Scientific) from 1 µg of total RNA with oligo(dT)_18_. Primer sequences were designed with Primer Quest and Oligoanalyzer software from the IDT web page (Table S1). Real-time PCR was performed using HOT FirePol EvaGreen qPCR Mix Plus (Solis BioDyne, Estonia) in an Eco Real-time PCR system (Illumina, USA). All qRT-PCR results were normalized with threshold cycle (Ct) values of the reference gene. The RQ value for the target gene was calculated as 2^−ΔΔCt^^33^. The values represent the mean of three biological replicates and two technical replicates.

### Statistical analysis

Physiological and biochemical data were subjected to analysis of variance (ANOVA). A two-way model was fitted. The model considered the fixed effects of water stress, tomato species and water stress × tomato species interaction. The comparison of means was made using Tukey tests in R software.

## Results

### Photosynthesis and biochemical and water relations change as a function of drought severity

**Figure 1:**
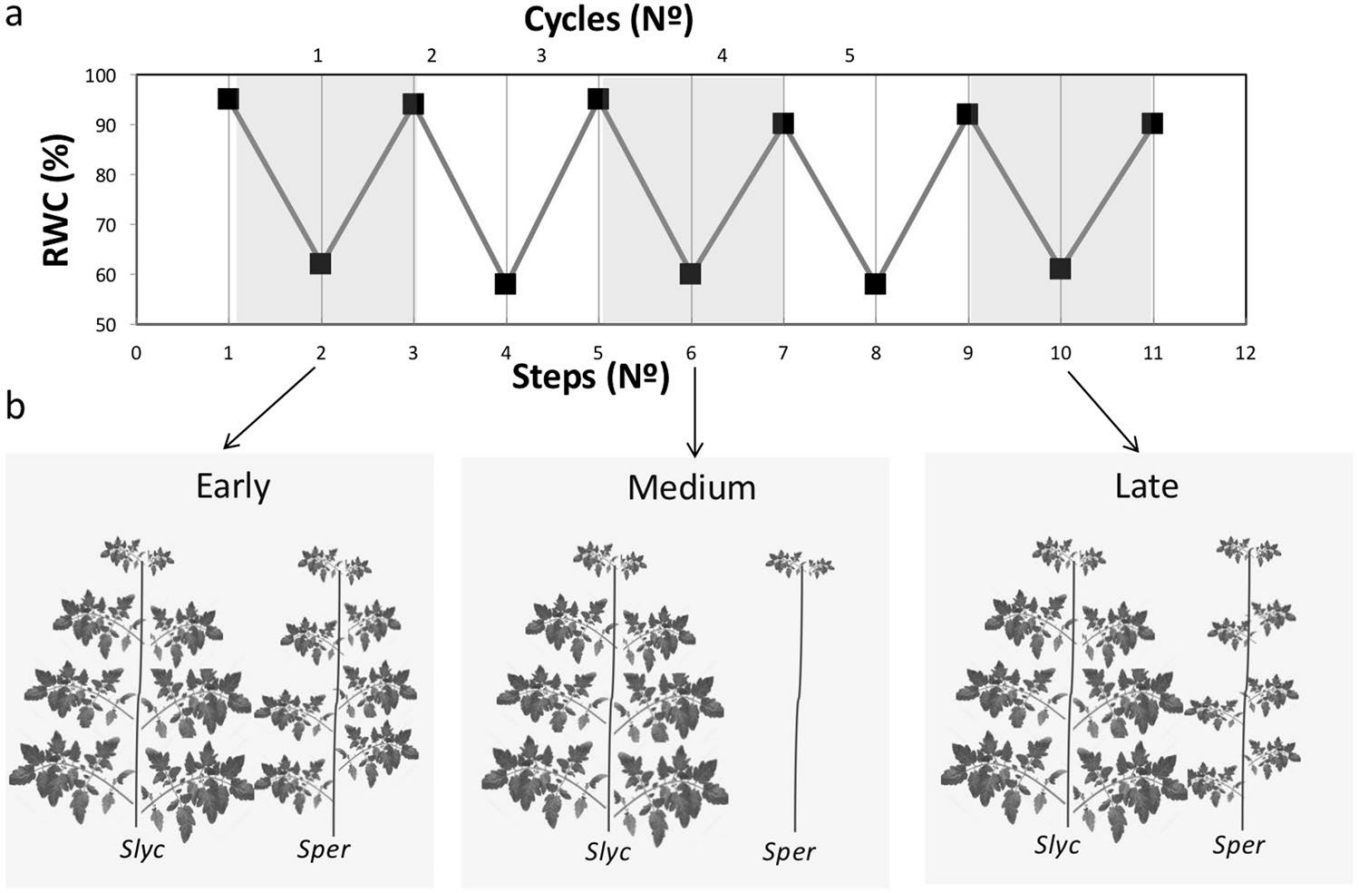
Representation of long-term water stress treatment and effect in *Sper* and *Slyc*. (**a**) Relative water content (RWC) under dehydration/rewatering (D/RW) cycles (grey and white sections) represented in three steps for cycle. (**b**) Plant differentiation between early-medium and late phase of morphological modification under drought treatments in *Slyc* and *Sper* then of several cycles of D/RW.

With the aim of evaluating the effect of water stress in *Sper* and *Slyc,* we performed a water stress treatment mediating the dehydration/rewatering cycle (D/RW cycles) (Fig. 1a). When the plants were exposed to the first D/RW cycle, no phenotypical changes were observed compared with the untreated plants; however, after 2–3 D/RW cycles in *Sper*, the lower mature leaves fell, while the shoots were maintained without obvious changes. In contrast, in *Slyc,* the leaves did not fall and remained on the plant for a long period. However, after five D/RW cycles, *Sper* was able to activate the axillary meristem and develop new leaves. *Slyc* maintained the same plant architecture without growth (Fig. 1b).

**Figure 2:**
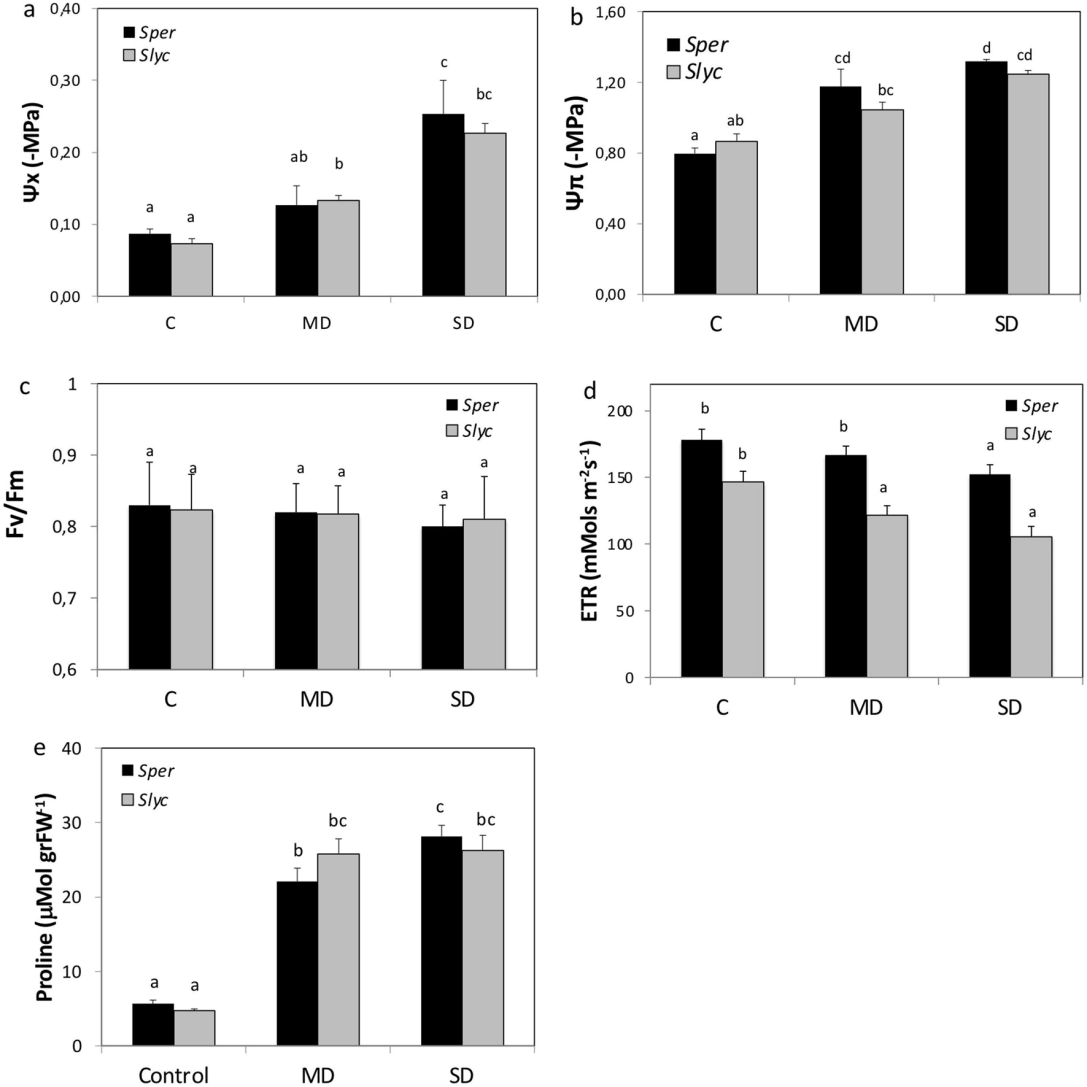
Physiological analysis of *Slyc* and *Sper* leaves under moderated and severe drought stress. (**a**) Water potential, (**b**) osmotic potential, (**c**) Chlorophyll fluorescense and (**d**) electron transport rate (photosynthesis), (**e**) proline concentration, for leaves of *Sper* (black bars) and *Slyc* (grey bars) under optimal (Control), moderated (MD) and severe (SD) drought treatment. Results are expressed as mean ± SE. Data with different letters across water stress and tomato species indicate significant differences according Tukey statistical test (*P* ≤ 0.05).

The differential behavior between the species suggested that a specific response activated during an early period occurred in *Sper.* We decided to investigate in detail the processes that occur in this early phase. Here, the D step shown in Fig. 1a (from the D/RW cycle) was performed to mediate a short-term water stress treatment using PEG 8000, considering two severity conditions, moderate (5% PEG, MD) and severe drought stress (12% PEG, SD), compared with plants irrigated only with water (C). Physiological data were registered in the early 24 h of the treatment. Under MD, no visible changes were observed in the plants. However, SD treatment plants began wilting after 24 h. The water potential was significantly increased in the absolute value under MD and SD compared with C. Additionally, a significant increase was observed from MD to SD in *Sper* (Fig. 2a). Similarly, the osmotic potential was increased from C to MD in *Sper* and from C to SD in *Slyc*. However, on average, both species responded proportionally to the drought severity (Fig. 2b). Photosynthetic system integrity was evaluated by mediating the measurement of Fv/Fm. This parameter did not change significantly between treatment and species, suggesting that no damage occurred during an early phase of drought stress (Fig. 2c). However, the electron transport rate (ETR) was higher in *Sper* than in *Slyc* under optimal irrigation. The ETR decreased proportionally to the severity, although this value was maintained higher in *Sper* than in *Slyc* (Fig. 2d).

The accumulation of proline was measured in fresh leaves. Figure 2e shows that the proline concentration increased 4 to 5 times during both drought treatments in *Slyc* and *Sper,* but no significant differences were observed between the species. No significant changes were observed between the treatments in *Slyc*. However, a significant increase was observed from MD to SD in *Sper* (Fig. 2e).

### RNA sequencing and differentially expressed genes (DEGs) in response to the species and treatment

To identify the genes that are activated or repressed between both tomato species in response to water stress, RNAseq analysis was performed for all treatments (C, MD and SD). An average of 14,346,942 raw reads were generated per library. In addition to trimming the adapter sequences, all reads with a mean quality value (Q) minor of 30 were removed (low-quality reads), retaining an average of 12,656,753 (88%) clean reads per library. An average of 92% of the clean reads were mapped to the tomato reference genome (Table S2). For each library, an average of 17,909 expressed and annotated genes were obtained, with averages of 17,602 and 18,217 for *Sper* and *Slyc*, respectively (Table S2).

To determine significant gene expression differences between libraries, a DEG test was performed using the following criteria: false discovery rate FDR < 0.05 and Log_2_(FC) > 2. A total of 7059 genes showed significant differences among all libraries. *Sper* showed an increase in significant gene expression (induced and repressed genes) according to the drought stress magnitude, and fourfold DEGs were observed in SD-*Sper* over MD-*Sper* treatment (Tables S3 and S4). On the other hand, in the *Slyc* species, the major significant gene expression was observed in the MD-*Slyc* treatment, which was 3.7-fold greater than that in the SD-*Slyc* treatment (Tables S3 and S4). Finally, differentially expressed genes of each species in response to drought stress were analyzed. In the *Sper* stress treatment, relative to the control treatment, C-*Sper* 1817 and 896 differentially expressed genes were identified as induced and repressed, respectively. On the other hand, in response to drought stress treatment, *Slyc* showed 1108 and 875 DEGs induced and repressed, respectively.

#### Functional analyses and GO enrichment

**Figure 3:**
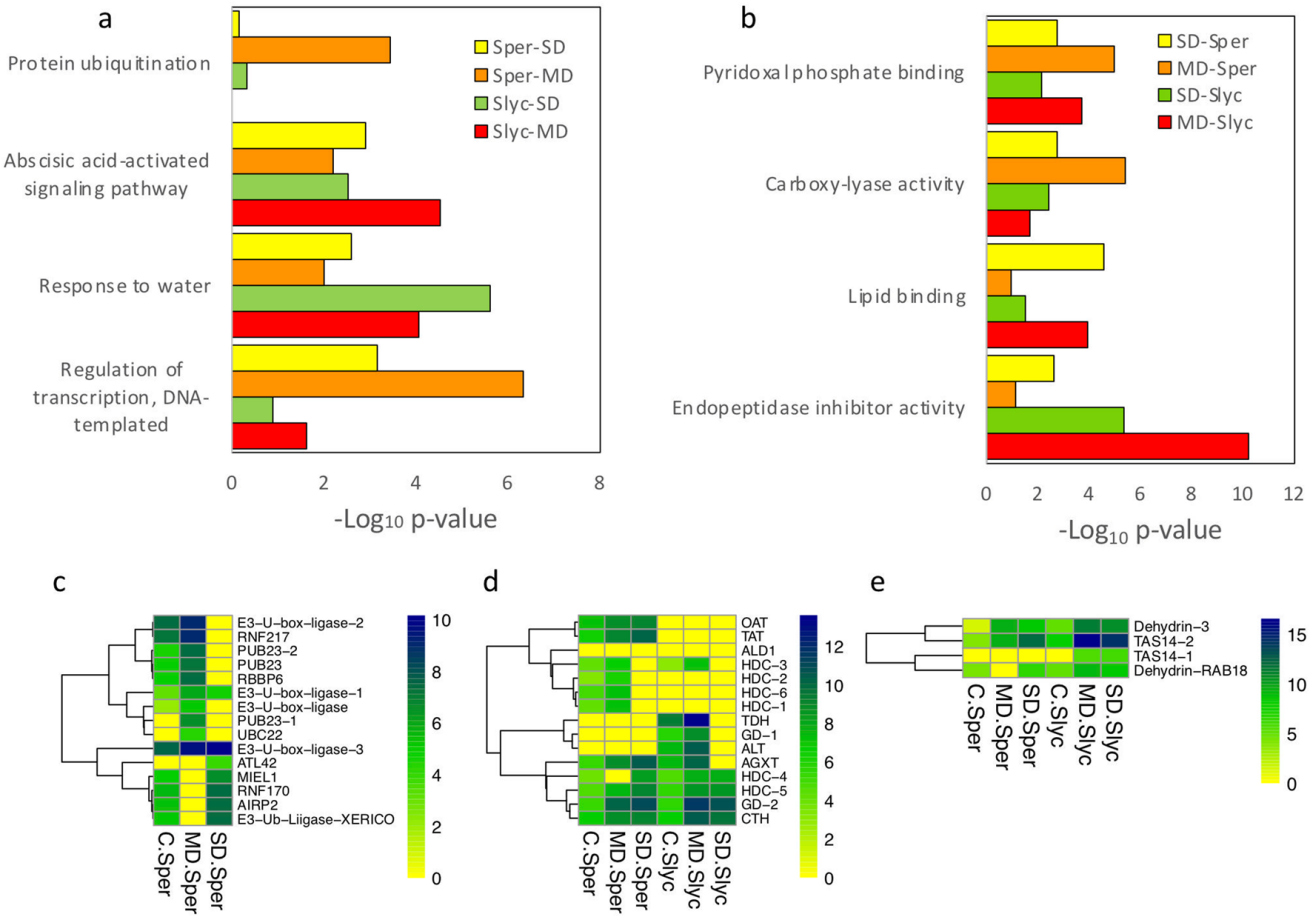
Gene Ontology functional classification for induced DEGs. Functional classification according categories of (**a**) biological process and (**b**) molecular function for group of DEGs corresponding to MD-*Sper*, SD-*Sper*, MD-*Slyc* and SD-*Slyc*. Selected GO subclasses were selected according the smaller *p*-value and non-redundant relationship according enrichment analysis using fisher exact test. Heatmap for expression of DEGs belong to Log_2_(Count + 1) for subclassifications: (**c**) protein ubiquitination; (**d**) piridoxal phosphate binding and e. respose to water.

**Figure 4:**
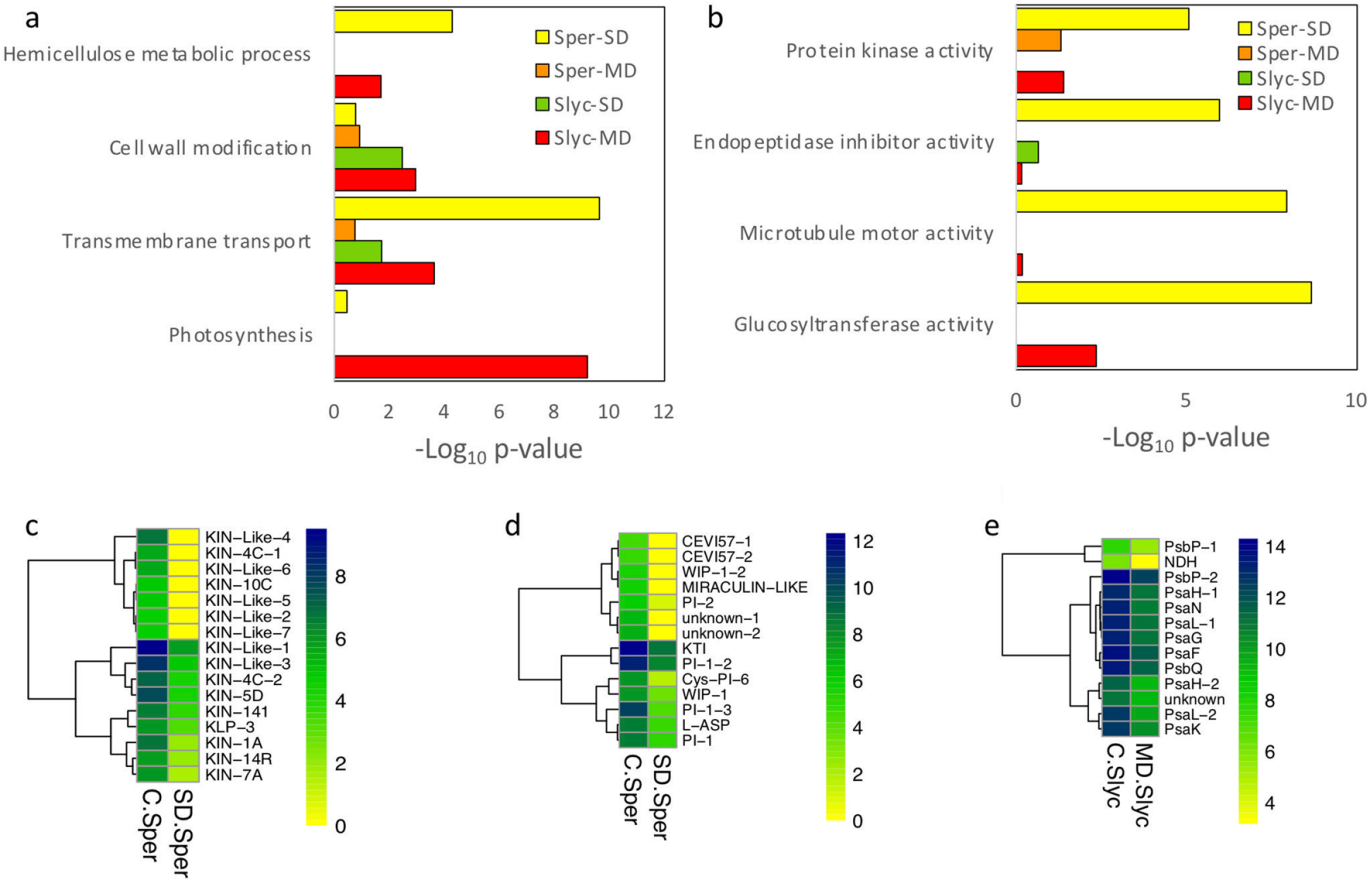
Gene Ontology functional classification for repressed DEGs. Functional classification according categories of (**a**) biological process and (**b**) molecular function for group of DEGs corresponding to MD-*Sper*, SD-*Sper*, MD-*Slyc* and SD-*Slyc*. Selected GO subclasses were selected according the smaller *p*-value and non-redundant relationship using enrichment analysis with fisher exact test. Heatmap for expression of DEGs belong to Log_2_(Count + 1) for subclassifications: (**c**) microtubule motor activity; (**d**) endopeptidase inhibitor activity and e. photosynthesis.

A summary of GO enrichment analysis was made using ShinyGO for the product of four main comparisons. Here, the 30 most low *p*-value categories of BP and MF were described as hierarchical clustering (Fig S1). A detailed analysis that included the most relevant categories that considered the requirement of the lowest *p*-value in nonredundant GO enrichment analysis was plotted for MD-*Sper*, SD-*Sper*, MD-*Slyc* and SD-*Slyc* DEGs separately for induced and repressed gene groups (Figs. 3 and 4). The category “protein ubiquitination” from BP was enriched in MD-*Sper*, which was composed of E3 ubiquitin ligase genes (E3s) (Fig. 3c). While, “pyridoxal phosphate binding” was enriched in all treatments and it was composed by genes related with aminoacid metabolism (Fig. 3d). The “lipid binding” category was more significantly enriched in SD-*Sper* and MD-*Slyc* (Fig. 3b, Table S5), while “regulation of transcription, DNA-templated” was highly enriched in MD-*Sper* but also in SD-*Sper* (Fig. 3a). The categories “endopeptidase inhibitor activity” from MF and “response to water” from BP were important in MD-*Slyc* and SD-*Slyc*, respectively (Fig. 3a,b). The category “response to water” was composed of known TAS14 and RAB18 dehydrins (Fig. 3e). The enrichment of “protease inhibitor proteins” in *Slyc* was between the most significant inductions compared with *Sper*. Other categories, such as “abscisic acid-activated signaling pathway” and “carboxy-lyase activity”, were also important (Fig. 3a,b, Table S5).

Between the GO categories significantly enriched in repressed DEGs were “microtubule motor activity”, glycosyltransferase activity”, “endopeptidase inhibitor activity” and “protein kinase activity”, “carbohydrate metabolic process” for SD-*Sper*, while “photosynthesis” was enriched in MD-*Slyc* (Fig. 4a,b). Some of these categories are described in detail in Fig. 4c,d,e.

#### Single and shared DEG groups distinguished between the tomato species

**Figure 5:**
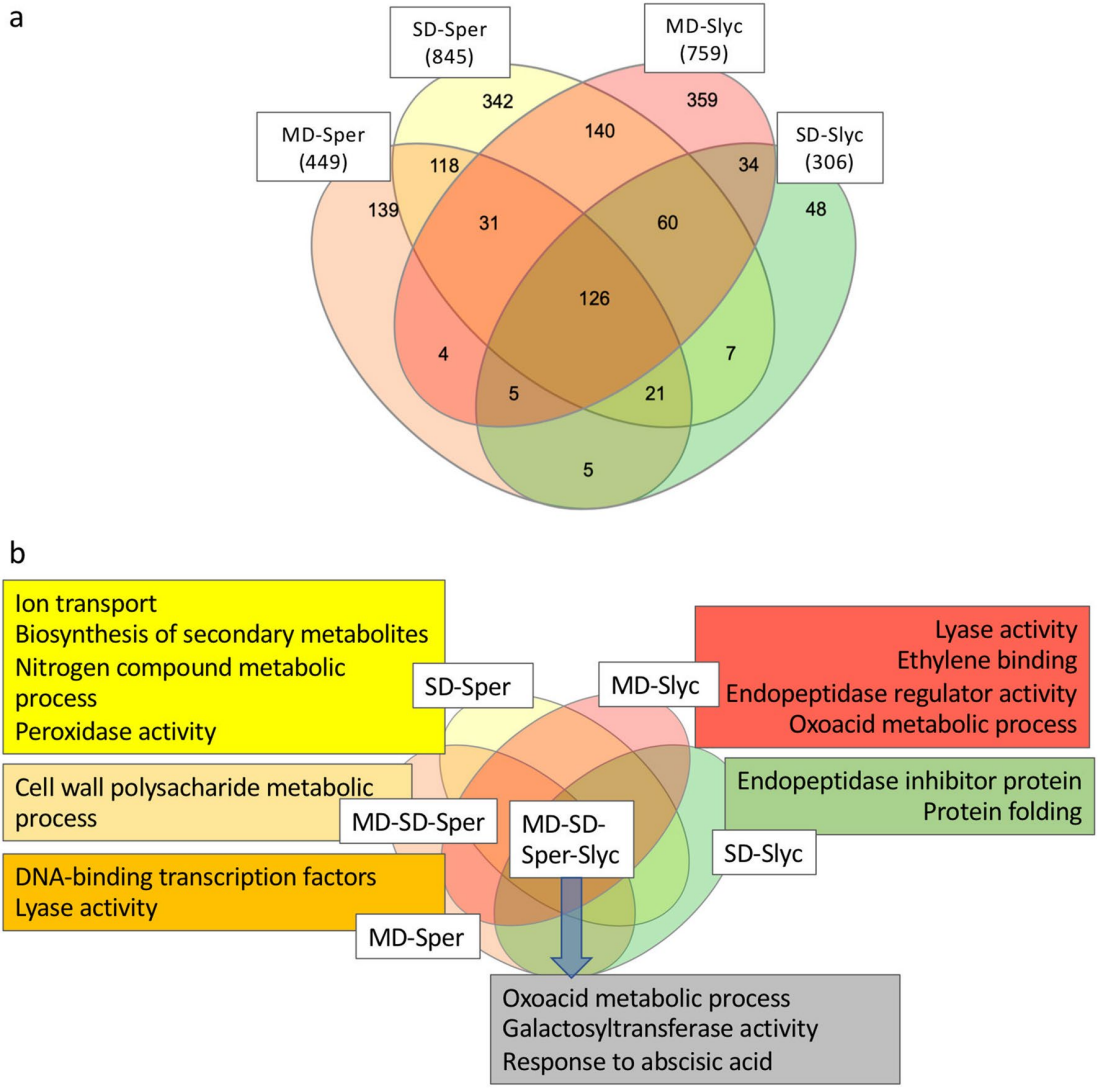
DEGs ordering and GO categories enrichment according specie and drought treatment. (**a**) Four way venn diagram for induced DEGs was made between MD and SD treatments in *Sper* and *Slyc*. (**b**) The most representative GO categories for each subgroup from venn diagram were outlined considering the lowest *p*-value for enrichment and redundancy.

**Figure 6:**
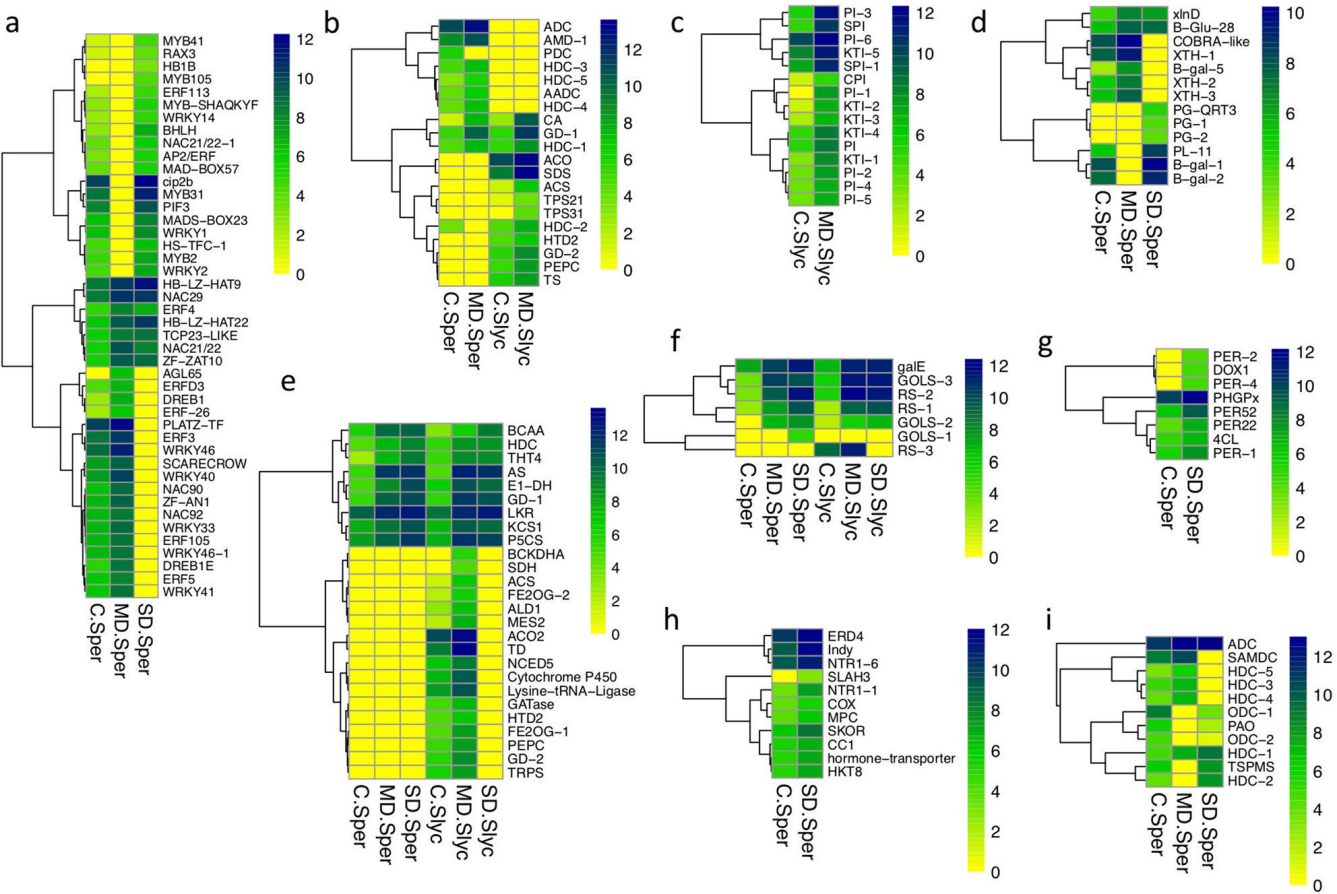
Gene expression of GO categories enriched in subgroups from induced DEGs. Heatmap for expression of DEGs belong to Log_2_(Count + 1) for subclassifications: (**a**) transcription factor; (**b**) lyase activity, (**c**) endopeptidase inhibitor activity; (**d**) Cell wall polysacharide metabolic process; (**e**) Oxoacid metabolic process; (**f**) Galactosyltransferase activity; (**g**) Peroxidase activity; (**h**) Ion transport; (**i**) Polyamines. Heatmap show row clustering using method ward.D2. Gene nomenclature and code are detailed in Table S5.

Among the induced and repressed DEGs, some were exclusively expressed in only one of the species, treatment or both, while it was also possible to find genes commonly induced in all of the treatments. In this research, a total of 449 genes were induced during MD-*Sper*, 139 of which were exclusive for this treatment (Fig. 5a). This subgroup was characterized by the inclusion of 18 genes coding for DNA-binding transcription factors, among which 5 WRKY, 5 ERF and 2 NAC family TFs were exclusively induced in the MD treatment (Figs. 5b, 6a).

The “cell wall polysaccharide metabolic process” was a shared category in *Sper* (Fig. 5b). Between them were β-xyloxidase (xlnD), polygalacturonases (PG), β-1,3-glucanases (β-glu), xyloglucan endotransglucosylase/hydrolase (XTH), pectate lyase (PL) and B-galactosidases (β-gal) (Fig. 6d).

**Figure 7:**
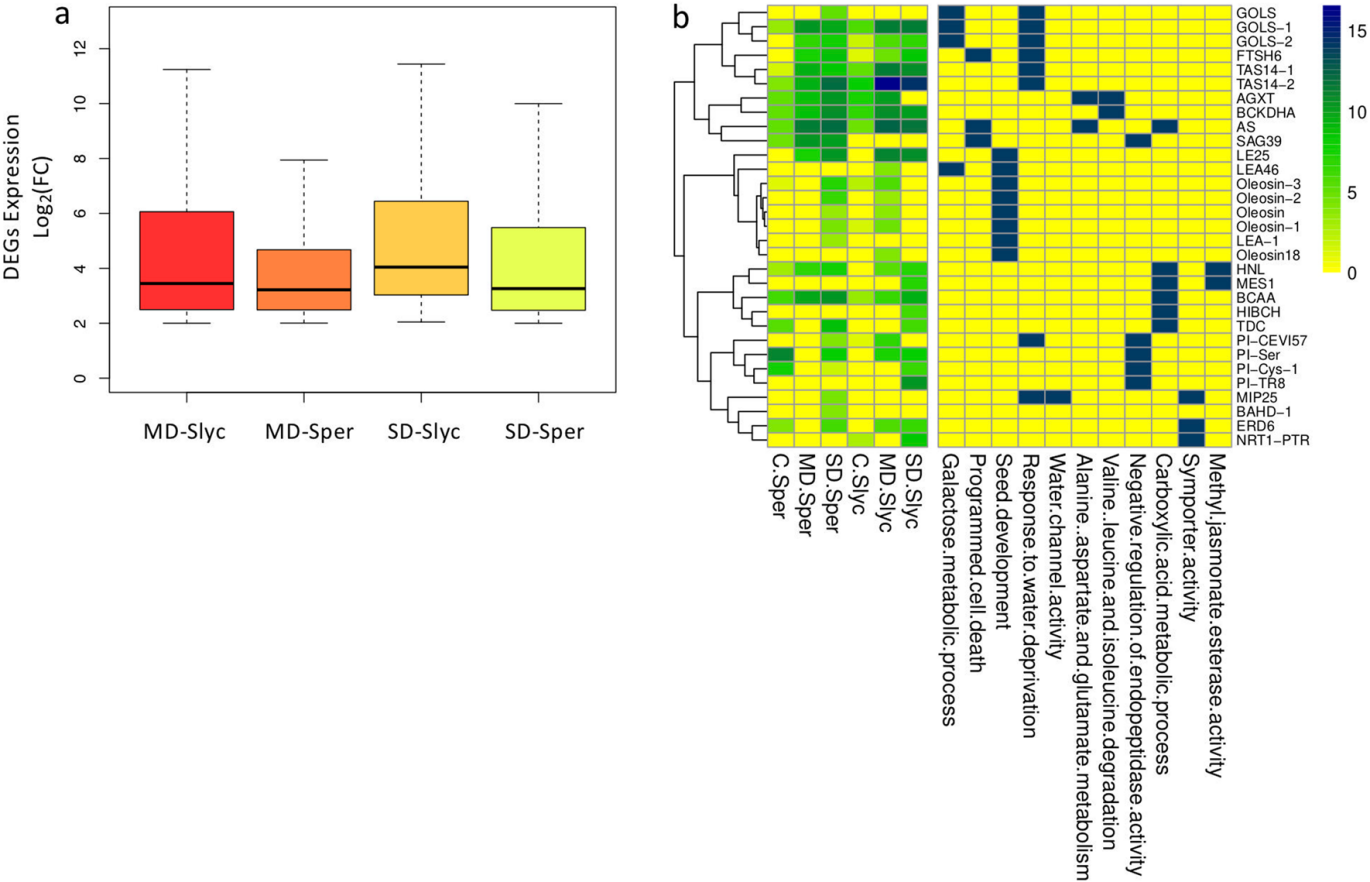
Induced DEGs distribution and most induced genes. Box plot for DEGs considering log_2_(FC) > 2 and FDR < 0.05. The median of Log_2_(FC) is represented by intermediate band from each box (**a**). The top whiskers from each comparison belonging to 25% most induced genes were appointed by GO categories (**b**). They were plotted in a heatmap according lowest *p*-value and showing expression value of called DEGs.

From 759 DEGs induced in MD-*Slyc*, many of the genes were exclusive to this treatment (Fig. 5a). Genes related to “lyase activity” were enriched in the MD-*Slyc* and MD-*Sper* subgroups. Here, hydrolyses and deaminase were exclusively from MD-*Slyc,* while histidine decarboxylase (HDC) and adenosyl methionine decarboxylase (AMD) were common in MD-*Sper* (Fig. 6b). Additionally, MD-*Slyc* was characterized by increased enrichment in endopeptidase regulator activity (PI), and ethylene binding (Fig. 5b). The PI group was composed of 14 genes coding for kunitz proteins, cysteine and serine-PI only induced in MD-*Slyc* (Fig. 6c). Similarly, several genes from “oxoacid metabolic process”, “galactosyl transferase activity” and “response to abscisic acid” were common for both treatments and species (Fig. 5b). The oxoacid metabolic process grouped genes related to amino acid metabolism, ABA and gibberellin biosynthesis (Fig. 6e). Some of these genes were exclusively induced in MD-*Slyc*, such as 9-cis-epoxycarotenoid dioxygenase (NCED5), threonine dehydratase (TD), and glutamate decarboxylase (GD). “Galactosyl transferase activity” included genes coding for galactinol synthase (GOLS) and raffinose synthase (RS) (Fig. 6f). Three GOLS genes were also grouped between the most induced genes in the transcriptome (Fig. 7b).

Some genes were grouped as “nitrogen compound metabolic process”, similarly than other categories such as “ion transport” and “peroxidase activity” enriched in SD-*Sper* (Figs. 5b, 6g,h) Here, we manually annotated some of them that were associated with polyamine biosynthesis (Fig. 6i), such as histidine decarboxylase (HDC), arginine decarboxylase (ADC), thermospermine synthase (TSPMS) and S-adenosylmethionine (SAM) decarboxylase (SAMdc).

From the four main comparisons, a total of 2337 genes were significantly repressed (Table S3). A total of 918 DEGs were exclusively repressed under SD-*Sper* (Fig. S2a). They were enriched in genes related to “mitotic cell cycle”, “endopeptidase inhibitor activity” and “glucosyl transferase activity” (Fig. S2b). Of 802 total genes repressed under MD-*Slyc,* only 375 genes were only repressed here. This selection was enriched in “photosynthesis”, “proline catabolic process” and “response to jasmonic acid” (Fig. S2b).

#### Enrichment from the fourth total most induced DEGs

Now, with the aim of determining the behavior of transcriptomes with respect to the global overexpression level, a boxplot was built. In general, nearly 50% of *Sper* DEG treatments showed a count number similar to or lower than the lowest 25% of *Slyc* treatments (Fig. S3). Additionally, the upper 25% of *Sper* DEGs had Log_2_(FC) values of 4.67–7.94 and 5.48–9.99 for MD/C and SD/C, respectively. While *Slyc* was between 6.06–11.24 and 6.44–11.43 for MD/C and SD/C, respectively (Fig. 7a). Although a positive increase in the dispersion of gene induction was observed in both species, during the SD treatments, the Log_2_(FC) value was higher in *Slyc* than in *Sper*. Therefore, we selected a subset composed of DEGs with Log_2_(FC) values over the third quartile from each comparison, and they were analyzed for GO (Fig. 7a,b). The GO analysis for the most relevant and nonredundant groups revealed new categories. Between them, we highlighted the new categories “seed development”, “methyl jasmonate esterease activity” and “programmed cell death”. From “seed development”, four genes coding for oleosin were induced in MD-*Slyc* or SD-*Sper*. Two genes coding for cysteine proteinase (SAG39) and a zinc metalloprotease (FTSH6) were highly induced under drought and related to “programmed cell death” (Fig. 7b). Additionally, aspartic proteinases, metaloendoproteinases and cysteine proteinases were specifically induced in MD-*Sper* or SD-*Sper* (Table S5). Genes related to “water channel activity” such as TIP3 and MIP25 and “symporter activity” for sugar (ERD6) and nitrogen transport (NRT1) were highly expressed in the drought treatments (Fig. 7b). Additionally, other transporters for sugar, nitrogen, metals and oligopeptides were induced in SD-*Sper* treatments (Fig. 6h).

Genes related to catabolism of branched-chain amino acids (valine, leucine and isoleucine), such as oxoisovalerate dehydrogenase (BCKDHA) and alanine glyoxylate aminotransferase (AGXT), were induced in both species. Additionally, the genes related to the metabolism of alanine, glutamate (Glu) and aspartate, such as AGXT in peroxisomes, asparagine synthetase (AS) and GD, were equally induced in both tomato species (Fig. 7b).

### Transcriptome validation using real-time quantitative PCR

To validate transcriptome assembly, the quantitative real-time (qRT-PCR) expression patterns of nine random genes representing different GO categories corresponded with the RNAseq expression data (Fig. S4).

## Discussion

The angiosperms have evolved and colonized the earth. Different extreme habitats, such as deserts, are an interesting focus of attention for understanding the evolution and diversification of herbaceous plants^34^. Solanum species are adapted to several environmental conditions^35^. Previously, our team suggested that constitutive adaptations through acclimation mediated by water restriction in desert tomato species play an important role in tolerance to drought stress. Here, the plant architecture restructuring and plasticity of wild tomatoes appear to be important traits for tolerating water restriction^8^.

In this research, we evaluated the transcriptomic response under equivalent conditions for two contrasting tomato species with respect to their drought tolerance. Here, the water and osmotic potential changes correlated with the severity of water stress treatment in both species (Fig. 2a,b). It activated the expression of known water stress marker genes such as Tas14 and RAB18 (Fig. 3e) in both wild and cultivated tomatoes. Both genes were previously identified in research on the early accumulation of abscisic acid in leaves during a short-term drought period in tomato^36, 37^.

The early drought treatment revealed some similarities in the photosynthesis response (Fv/Fm) in both species (Fig. 2c). Similar results at an early time have been obtained in maize and Arabidopsis under several days of drought treatment^38, 39^.

An increase in osmotic potential is a common response under plant water stress and is related to sugar accumulation in tomato^40, 41^. Susy and alkaline/neutral invertase genes involved in sucrose metabolism, GOLS and RAFS related to RFO biosynthesis were induced in *Sper* and *Slyc* (Fig. 6f, Table S5). Sugars accumulate under drought, acting as osmolytes to maintain cell turgor and stabilize cell proteins, but also suggested to protect plant cells from oxidative damage^42–45^.

Additionally, water in plant tissues controls turgor pressure and directly affects the extensibility of the cell wall. Glycosyl hydrolases promote the hydrolysis of cell wall polysaccharides and provide a mechanism to modify their rigidity and subsequently control their volume^46^. The upregulation of genes related to cell wall polysaccharide metabolic processes in *Sper* can contribute to regulating the cell volume under adverse conditions to contribute to osmotic regulation under water stress (Fig. 2a, 6d).

Similarly, the amino acid proline plays a role as an osmotic regulator during drought stress and also stabilizes the subcellular structure, energy sink and scavenger activity of subcellular structures^39^. Here, an increase in proline was detected at 24 h of drought stress (Fig. 2e) and was correlated with the induction of the OAT and P5CS genes and downregulation of ProDH, which were previously described to be involved in the control of proline accumulation (Fig. 6e, Table S5)^47, 48^.

Additionally, several changes in the expression of genes related to amino acid metabolism were shown in both species as an effect of water stress. Transaminases such as alanine, aspartate and tyrosine aminotransferases, in addition to AS, SGSD and P5CDH, promote the accumulation of Glu. In this research, some of them were grouped in the “piridoxal phosphate binding” and “oxoacid metabolic process" GO groups and enriched without many differences between the species (Figs. 3d and 6e). Glu occupies a central position in amino acid metabolism in plants. It is the precursor of several amino acids, glutathione and GABA. Additionally, the different GDs induced under water stress in this research suggest the conversion of Glu to GABA. Glu and GABA accumulate in plants under abiotic stress and promote tolerance^49, 50^. In Arabidopsis, an increase in Glu was reported just after the onset of senescence^51^. Additionally, GABA has been shown to be involved in the recycling and reallocation of nitrogen during leaf senescence^52^, suggesting that in tomato species, a similar process can occur.

Although nitrogen is the element predominantly remobilized during leaf senescence, other research shows that many other elements are also transported, although less efficiently than nitrogen^53^. Ion transport GO category was enriched in *Sper* (Fig. 5b). Proteins involved in the transport or storage of iron (ferritin), nitrogen (NRT1/PTR family), amino acids, ABC type or metal ion transporters were represented and may illustrate the importance of the remobilization of nitrogen and valuable metal ions (Fig. 6h).

TFs were highly enriched under some conditions of water stress in this research (Figs. 3a, 5b). They belong mainly to the WRKY, NAC, ERF and MYB TF families (Fig. 6a). The TFs SlORE1S03 and SlORE1S06 are orthologous to AtORE1 from Arabidopsis, which encodes a master regulator of senescence initiation^54–56^. SlORE1S03 and SlORE1S06 were induced in Md-*Sper* and MD-*Slyc,* respectively. NAC TF nonripening (NOR), recently described by Ma et al.^57^ as a controller of leaf senescence, was induced here in *Slyc* and *Sper*. However, others such as WRKY46, WRKY41 and NAC29 TFs were induced only in MD-*Sper* (Fig. 6a, Table S5). They are homolog with TFs related to leaf senescence, such as WRKY53 and ANAC029^57, 58^. These results suggest that MD and SD treatment activate signaling to senescence mediated by common and differentially expressed TFs.

On the other hand, ubiquitination is a prevalent posttranslational modification system. It involves the attachment of ubiquitin to selected substrates targeting modified proteins to the multiproteolytic 26S proteasome complex for degradation. E3 ubiquitin ligases (E3s) play a major role in recruiting target proteins for ubiquitination. E3s have emerged as modulators of plant responses to drought between other abiotic stresses^59, 60^. In *Sper*, three homologous genes to E3s PUB23 were induced under MD. Additionally, homologous MIEL1 and XERICO E3s were upregulated in SD (Fig. 3c). The PUB23 gene functions as a negative regulator in the water stress response in Arabidopsis. XERICO activates ABA synthesis and improves tolerance to drought stress, while MIEL1 negatively regulates wax accumulation^61–63^. Considering the previous evidence, ubiquitination promotes differential regulation under the early drought response in *Sper*, but it also depends on the water stress severity.

On the other hand, during abiotic stress, plants can also downregulate their cell division process, since some plants have adapted quiescent mechanisms to survive desiccation. Mitotic cell division can be repressed depending on drought severity by arresting in G1/S or the G2/G transition phases^64–66^. Cyclin-dependent protein kinases (CDKs) and cyclins are regulators of the cell cycle. During this research, “mitotic cell cycle” and “microtubule motor activity” groups were repressed specifically in SD-*Sper* (Fig. 4b, Fig. S2). They mainly coded for cyclin and cytoskeletal kinesin protein genes (Fig. 4c, Table S5). This decrease implies that cell division and growth were suppressed during SD in *Sper*.

Most relevant genes considering the highest Log_2_FC in this research included those related to the development of seeds and also the response to water deprivation and senescence (Fig. 7). Between them, oleosin provides stability, preventing abnormal fusion of oil bodies during seed desiccation. An increase in oleosin in leaves under drought stress can enable the long-term accumulation of TAG^67^. Other significantly induced genes were PI, mainly in *Slyc* (Figs. 3b, 5b, 6c, 7b). However, induction was also observed for proteinases (Fig. 7b, Table S5). Among them, the senescence-specific cysteine protease SAG39, reported previously in rice, was upregulated by water stress in this study. The regulation of proteolysis is vital for plant cell survival under stress. Protease inhibitors appear to be regulators of endogenous proteolysis during water stress in *Slyc,* maintaining leaf integrity and delaying leaf senescence^68–74^.

The low induction of PI in *Sper* can suggest an uncontrolled and accelerated senescence process during early water stress episodes. One of the main symptoms of senescence is the repression of photosynthesis genes (Fig. 4a,e). However, in contrast to *Slyc, Sper* did not repress genes associated with photosynthesis. In this sense, although the electron transport rate in PSII decreased with the severity of water restriction, the highest reduction was in *Slyc* (Fig. 2d). During early leaf senescence, an increase in protease activity has been reported. However, the exogenous application of polyamines retards this process by decreasing protease activity and chlorophyll loss^75^. In this research, genes related to polyamine biosynthesis were upregulated in *Sper*. They coding for HDC, ADC, TSPMS, and S-adenosylmethionine (SAM) decarboxylase (SAMdc) (Fig. 6i).

PAs regulate several processes in plants, including senescence, programmed cell death (PCD), and homeostatic adjustments in response to external stimuli and stresses. Additionally, PAs participate in the control of the N:C balance^76^. The N flow toward PAs may serve as a strategy of cells to store N^77^. The accumulation of PAs during abiotic stress prevents membrane damage and protein degradation in chloroplasts, stabilizing chlorophyll-associated complexes. Additionally, they act as free radical scavengers^78^. Studies in *Solanum chilense*, a near relative of *Sper*, showed that salt stress induced an increase in spermine accumulation, contributing to salt tolerance^79^. However, the disruption of PA homeostasis during abiotic stress also triggers senescence processes. This is a consequence of PA oxidases that generate H_2_O_2_^80, 81^. The results showed the upregulation of PA gene biosynthesis and the downregulation of PA oxidase in *Sper* but not in *Slyc* (Fig. 6i). This evidence suggests that activation of polyamine biosynthesis in *Sper* can provide protection to cell structures in the leaves during the temporal water stress episode, delaying the senescence-triggered process.

### Conclusion

The present study addresses the earliest step of the acclimation process that triggers changes from optimal conditions of growth to a water restrictive state. Our research provides evidence about the differential regulation of gene expression between two contrasting drought-tolerant species and their relationship with some main physiological traits and also provides a glimpse of the consequences of this response on plant behavior. They are important because the changes that occur under early drought between both species trigger phenotypic changes during the middle and late phases (Fig. 1). Additionally, this fleeting transcriptomic response is just one part of the process of acclimatization of plant species from one environmental condition to another.

Drought induced accelerated senescence in *Sper*, but not in *Slyc*. In spite of some gene expression changes being conserved between wild and cultivated tomatoes under drought treatments, such as some genes related with stress, sugar accumulation or amino acid metabolism, also important differences were found. They can explain phenotypic changes observed between both species. Although some TFs related with senescence were induced in *Sper* and *Slyc*, others were exclusively induced in *Sper*. It is correlated with the induction of genes coding for ubiquitination process, ion transport and proteinases in *Sper*. Additionally, here is observed an induction absence of PI proteins. Although it can appear an uncontrolled senescence process, groups of genes such as the involved in PAs metabolic pathway were differentially regulated in *Sper*. It suggests a differentially protective role comparatively with *Slyc*, where PI proteins can allow the maintenance of leaf tissue during the drought stress.

Here, we suggest that the *Sper* response is associated with perennial character and phenotypic plasticity. Under an environmental change, *Sper* prefer adequate the leaf architecture to new conditions mediating an early and efficient recycling of resources and senescence. Although the transcriptomic response is conserved for a group of genes, changes that are triggered from an early phase define two different pathways for both tomato species. These are opportunities for future successful manipulation of genomes to generate stress-tolerant crops.

## Supplementary Information

Supplementary Figure S1.

Supplementary Figure S2.

Supplementary Figure S3.

Supplementary Figure S4.

Supplementary Legends.

Supplementary Tables.

## Data Availability

All data needed to evaluate the conclusions in the paper are present in the paper or in the Supplementary Materials.
