# Supplementary material for: Early transcriptional responses in Solanum peruvianum and Solanum lycopersicum account for different acclimation processes during water scarcity events
Source: Sci Rep. 2021 Aug 5;11:15961. doi: 10.1038/s41598-021-95622-2 (PMC8342453; doi:10.1038/s41598-021-95622-2)

Fig S1: A hierarchical clustering tree summarizing the correlation among significant GO categories .

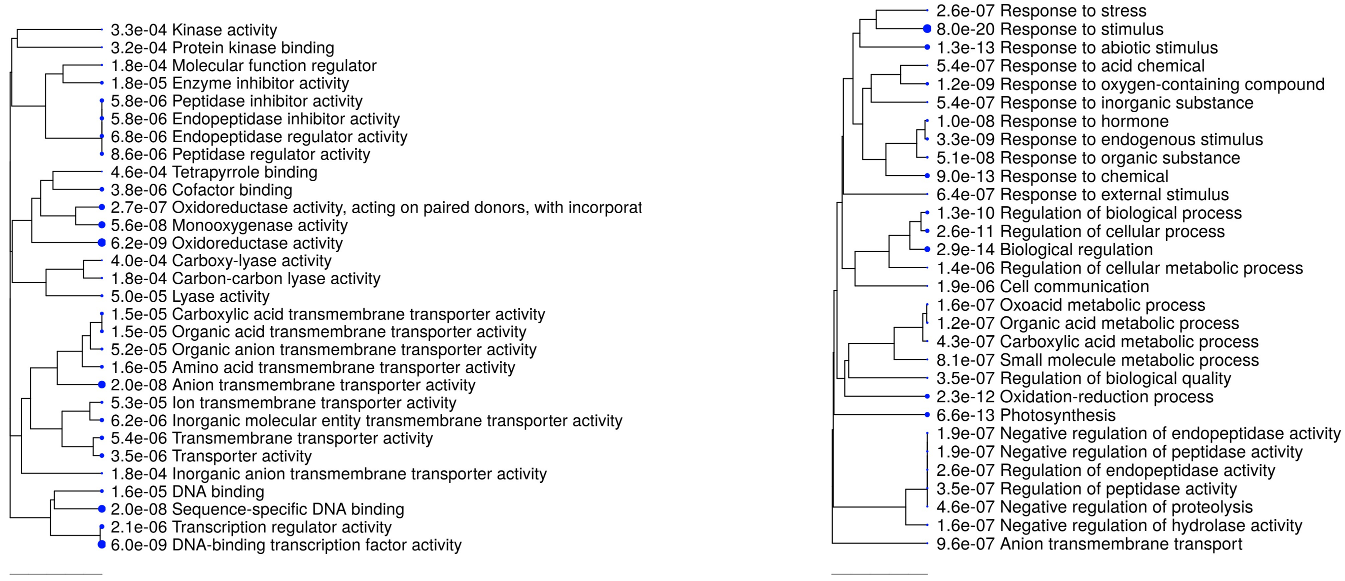

Supplement: Supplementary file 1 — Supplementary Figure S1. [file 41598_2021_95622_MOESM1_ESM.pdf]
