# Supplementary figures and images for: Early transcriptional responses in Solanum peruvianum and Solanum lycopersicum account for different acclimation processes during water scarcity events
Source: Sci Rep. 2021 Aug 5;11:15961. doi: 10.1038/s41598-021-95622-2 (PMC8342453; doi:10.1038/s41598-021-95622-2)

Fig S3. Count number from total DEGs. Boxplot for count number for DEGs from each treatment.

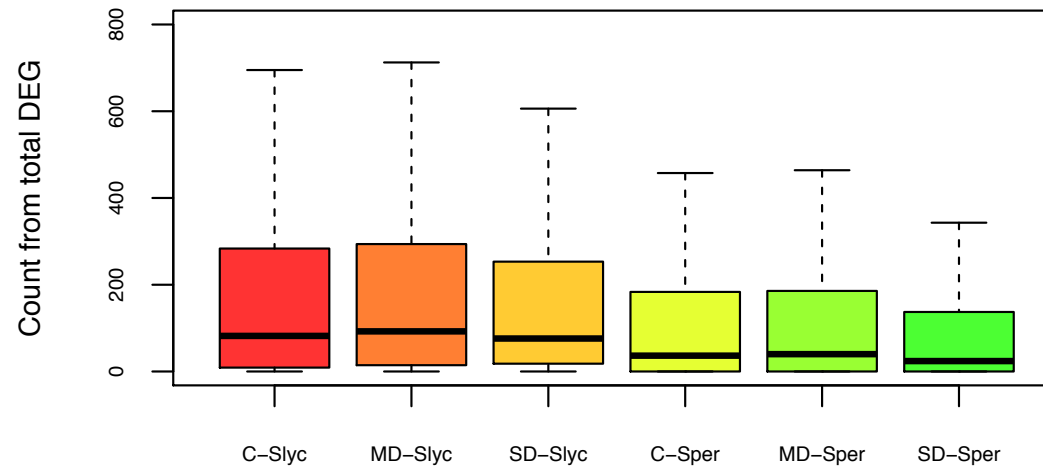

Supplement: Supplementary file 3 — Supplementary Figure S3. [file 41598_2021_95622_MOESM3_ESM.pdf]

Fig S4: Validation of transcriptomic RNaseq analysis for eight representative DEGs

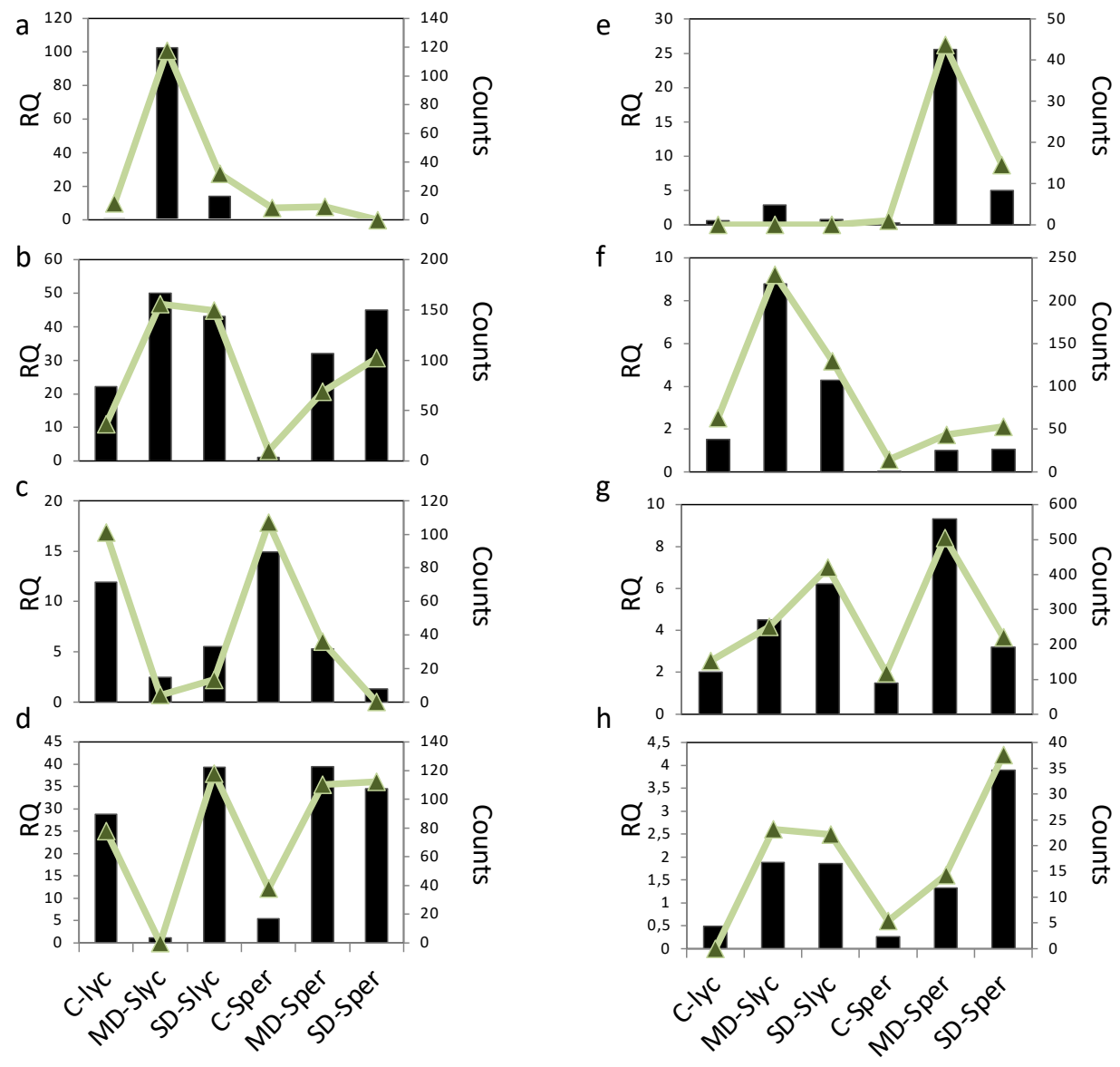

Supplement: Supplementary file 4 — Supplementary Figure S4. [file 41598_2021_95622_MOESM4_ESM.pdf]
