# Supplementary material for: Early transcriptional responses in Solanum peruvianum and Solanum lycopersicum account for different acclimation processes during water scarcity events
Source: Sci Rep. 2021 Aug 5;11:15961. doi: 10.1038/s41598-021-95622-2 (PMC8342453; doi:10.1038/s41598-021-95622-2)
Supplement: Supplementary file 5 — Supplementary Legends. [file 41598_2021_95622_MOESM5_ESM.pdf]

Early transcriptional responses in *Solanum peruvianum* and *Solanum lycopersicum* account for different acclimation processes during water scarcity events

Tapia G[1]*., González M[2]., Burgos J[1]., Vega MV[1]., Méndez J[1]., Inostroza L[1].

[1]Unidad de Recursos Genéticos Vegetales, Instituto de Investigaciones Agropecuarias, INIA-Quilamapu, Avenida Vicente Mendez 515, Chillán, Chile.

[2]Laboratorio de Microbiología Aplicada, Centro de Estudios Avanzados en Zonas Áridas (CEAZA). Raúl Bitrán 1305, La Serena, Chile.

Corresponding author: *gtapia@inia.cl

Supplementary material section

Figure S1: A hierarchical clustering tree summarizing the correlation among significant GO categories. GO categories for a. Molecular function and b. biological process. Pathways with many shared genes are clustered together. Bigger dots indicate more significant P-values.

Figure S2. DEGs ordering and GO categories enrichment according specie and drought treatment for repressed genes. a. Four way venn diagram for repressed DEGs was made between MD and SD treatments in *Sper* and *Slyc*. b. The most representative GO categories for each subgroup from venn diagram were outlined considering the lowest p-value for enrichment and redundancy.

Figure S3. Count number from total DEGs. Boxplot for count number for DEGs from each treatment.

Figure S4. Validation of transcriptomic RNAseq analysis for eight representative DEGs. Comparison of gene expression obtained by RNAseq (green line) and qRT-PCR (black bars). a. PPO, b. LEA, c. OXO, d.ATJ11, e.UnKn, f. LTPM, g. GLA, h. ECH. Tomato CAC, UBI and EF1α were used as internal standard. The qRT- PCR values correspond to three biological replicates and the error bars to standard deviation.
