# Supplementary material for: Early transcriptional responses in Solanum peruvianum and Solanum lycopersicum account for different acclimation processes during water scarcity events
Source: Sci Rep. 2021 Aug 5;11:15961. doi: 10.1038/s41598-021-95622-2 (PMC8342453; doi:10.1038/s41598-021-95622-2)
Supplement: Supplementary file 6 — Supplementary Tables. [file 41598_2021_95622_MOESM6_ESM.docx]

**Table S1.**Primer sequences

| **Gene primer** | **Primer sequence** |
| --- | --- |
| **PPO-F** | AAGGACTAGAAGTGGAGAGTGA |
| **PPO-R** | CTTCCAGCAAACTCAGCATTATC |
| **LEA-F** | GCTCAGGCAACCCATGATA |
| **LEA-R** | GCTGCTCCACTTGTCTTATCT |
| **OXO-F** | CCCAGTTTCCAGGTCTTAACG |
| **OXO-R** | ACCAGGGTGAGTGTGAAATG |
| **Unk-F** | AGCAAGGGAGGTTATTGTTGT |
| **Unk-R** | ATCGCCGACGACGTAGATA |
| **LTPM-F** | GAGGCTGTTGTGGTGTCATTA |
| **LTPM-R** | AGAAAGACCAGCAGCTTTACC |
| **ECH-F** | AGAGAAGGACGCAAGCAAA |
| **ECH-R** | CCACCCTCATAGAAATCGTTGTA |
| **GLA-F** | CCTTAATGCTACTGGACGAACT |
| **GLA-R** | GGCAATCGTTGTCATGCTTATC |

| **Table S2**. Summary of the sequencing, pre-processing and genome reference assembly. | | | | | | | | |
| --- | --- | --- | --- | --- | --- | --- | --- | --- |
| **Samples** | **Rep** | **Raw reads** | **Clean reads (Q20)** | **Clean reads (Q25)** | **Clean reads (Q30)** | **GC% Content Clean reads (Q30)** | **Mapped Reads (%)** | **No. Mapped Genes** |
| C-Sper | 1 | 16,541,177 | 15,787,611 | 15,384,610 | 14,363,973 | 42 | 88.19 | 17,989 |
| C-Sper | 2 | 17,611,364 | 16,958,529 | 16,602,359 | 15,651,573 | 43 | 90.61 | 18,171 |
| MD-Sper | 1 | 13,558,030 | 13,034,295 | 12,752,773 | 12,003,883 | 42 | 90.35 | 17,595 |
| MD-Sper | 2 | 13,149,180 | 12,609,623 | 12,313,580 | 11,543,214 | 42 | 88.13 | 17,722 |
| SD-Sper | 1 | 13,584,658 | 13,035,829 | 12,734,365 | 11,957,529 | 42 | 87.93 | 17,447 |
| SD-Sper | 2 | 13,715,550 | 13,089,680 | 12,740,206 | 11,878,483 | 44 | 85.54 | 16,690 |
| C-Slyc | 1 | 14,522,724 | 13,976,932 | 13,676,484 | 12,885,863 | 42 | 97.28 | 18,328 |
| C-Slyc | 2 | 13,826,393 | 13,324,718 | 13,051,569 | 12,324,146 | 42 | 97.82 | 18,748 |
| MD-Slyc | 1 | 14,058,800 | 13,513,407 | 13,214,892 | 12,434,025 | 42 | 96.91 | 18,605 |
| MD-Slyc | 2 | 12,720,552 | 12,258,841 | 12,003,531 | 11,324,936 | 41 | 97.91 | 18,424 |
| SD-Slyc | 1 | 14,740,812 | 14,176,582 | 13,867,928 | 13,053,079 | 42 | 97.67 | 18,413 |
| SD-Slyc | 2 | 14,134,069 | 13,577,974 | 13,266,217 | 12,460,335 | 43 | 88.02 | 16,783 |
|  |  |  |  |  |  |  |  |  |

| **Table S3.** Significative (differentially expressed) induced genes among all the treatments. Each comparison is shown as a treatment in row A vs column B. | | | | | | | |
| --- | --- | --- | --- | --- | --- | --- | --- |
|  |  | **C-*Sper*** | **MD-*Sper*** | **SD-*Sper*** | **C-*Slyc*** | **MD-*Slyc*** | **SD-*Slyc*** |
|  | **C-*Sper*** |  | 51 | 1368 | 754 | 2236 | 459 |
|  | **MD-*Sper*** |  |  | 294 | 650 | 1135 | 1 |
|  | **SD-*Sper*** |  |  |  | 1223 | 1176 | 63 |
|  | **C-*Slyc*** |  |  |  |  | 802 | 306 |
|  | **MD-*Slyc*** |  |  |  |  |  | 42 |
|  | **SD-*Slyc*** |  |  |  |  |  |  |

| **Table S4.** Significative (differentially expressed) repressed genes among all the treatments. Each comparison is shown as a treatment in row A vs column B. | | | | | | | |
| --- | --- | --- | --- | --- | --- | --- | --- |
|  |  | **C-*Sper*** | **MD-*Sper*** | **SD-*Sper*** | **C-*Slyc*** | **MD-*Slyc*** | **SD-*Slyc*** |
|  | **C-*Sper*** |  | 449 | 845 | 1505 | 2243 | 952 |
|  | **MD-*Sper*** |  |  | 90 | 728 | 1037 | 1 |
|  | **SD-*Sper*** |  |  |  | 2411 | 2122 | 287 |
|  | **C-*Slyc*** |  |  |  |  | 759 | 116 |
|  | **MD-*Slyc*** |  |  |  |  |  | 82 |
|  | **SD-*Slyc*** |  |  |  |  |  |  |

**Table S5.** DEGs for main GO categories. Gene names including Solgenomics code and GO category.

| **Gene** | **Code** | **GO category** |
| --- | --- | --- |
| WSD1 | Solyc01g011430 | Transferase activitytransferase activity, transferring acyl groups other than amino-acyl groups |
| WSD1 | Solyc01g095930 |  |
| HT | Solyc02g062710 |  |
| KCS | Solyc02g063140 |  |
| ASAT | Solyc02g081740 |  |
| ASAT | Solyc02g081750 |  |
| ASAT | Solyc02g081760 |  |
| SAT | Solyc02g082850 |  |
| BADH | Solyc04g078660 |  |
| BTB/POZ-domain prot | Solyc06g071830 |  |
| ACT | Solyc06g074710 |  |
| WSD1 | Solyc07g053890 |  |
| THT4 | Solyc08g006730 |  |
| HCT | Solyc08g068690 |  |
| THT4 | Solyc08g068690 |  |
| THT1-3 | Solyc08g068720 |  |
| THT1-3 | Solyc08g068730 |  |
| No data | Solyc08g068770 |  |
| KCS | Solyc09g083050 |  |
| CER-26 | Solyc09g092270 |  |
| KCS | Solyc10g009240 |  |
| KCS | Solyc11g072990 |  |
| CER-2 | Solyc12g087980 |  |
| COBRA-like | Solyc01g065530 | Cell wall macromolecule metabolic process |
| xlnD | Solyc01g107090 |  |
| β-gal-1 | Solyc01g110000 |  |
| PG-1 | Solyc02g067650 |  |
| PG-QRT3 | Solyc02g068410 |  |
| βGlu-28 | Solyc02g086700 |  |
| PL-11 | Solyc02g093580 |  |
| β-gal-2 | Solyc03g121540 |  |
| XTH-1 | Solyc04g008210 |  |
| PG-2 | Solyc04g015530 |  |
| β-gal-5 | Solyc04g080840 |  |
| XTH-2 | Solyc07g056000 |  |
| XTH-3 | Solyc12g017240 |  |
| RS-1 | Solyc01g079300 | Galactosyltransferase activity |
| GOLS-1 | Solyc01g100830 |  |
| GOLS-2 | Solyc02g062590 |  |
| GOLS-3 | Solyc02g084980 |  |
| RS-2 | Solyc02g086530 |  |
| RS-3 | Solyc07g065980 |  |
| galE | Solyc08g082440 |  |
| XTH6 | Solyc01g107800 | hemicellulose metabolic process |
| XTH8 | Solyc02g091920 |  |
| unknown-1 | Solyc03g031800 |  |
| GH16-2 | Solyc03g093110 |  |
| XTH33 | Solyc03g093130 |  |
| XTH-2 | Solyc05g005680 |  |
| XTH9 | Solyc07g009380 |  |
| unknown-2 | Solyc07g052980 |  |
| GH16-1 | Solyc12g011023 |  |
| XTH-1 | Solyc12g011030 |  |
| XTH-3 | Solyc12g017240 |  |
| CC1 | Solyc01g104780 | Ion transport |
| COX | Solyc03g043850 |  |
| SKOR | Solyc05g051220 |  |
| SLAH3 | Solyc06g036440 |  |
| hormone-transporter | Solyc06g050790 |  |
| MPC | Solyc06g071890 |  |
| HKT8 | Solyc07g014690 |  |
| ERD4 | Solyc08g023440 |  |
| Indy | Solyc11g012360 |  |
| NTR1-6 | Solyc12g006050 |  |
| NTR1-1 | Solyc12g095810 |  |
| HB1B | Solyc10g085210 | Regulation of transcription, DNA-templated |
| TCP23-LIKE | Solyc11g020670 |  |
| NAC90 | Solyc11g068620 |  |
| HS-TFC-1 | Solyc12g007070 |  |
| ERF18 | Solyc12g009240 |  |
| ERF113 | Solyc12g042210 |  |
| MYB2 | Solyc01g057910 |  |
| cip2b | Solyc01g079870 |  |
| MAD-BOX57 | Solyc01g087990 |  |
| WRKY46 | Solyc01g095630 |  |
| SCARECROW | Solyc01g100200 |  |
| PIF3 | Solyc01g102300 |  |
| BHLH | Solyc01g106460 |  |
| ZF-ZAT10 | Solyc01g107170 |  |
| ERFD3 | Solyc01g108240 |  |
| WRKY14 | Solyc02g021680 |  |
| HB-LZ-HAT22 | Solyc02g063520 |  |
| WRKY2 | Solyc02g071130 |  |
| ZF-AN1 | Solyc02g087210 |  |
| HB-LZ-HAT9 | Solyc02g091930 |  |
| ERF105 | Solyc03g093550 |  |
| ERF105-1 | Solyc03g093560 |  |
| SlORE1S03 | Solyc03g115850 |  |
| MYB31 | Solyc03g116100 |  |
| WRKY40 | Solyc03g116890 |  |
| AP2/ERF | Solyc03g117720 |  |
| DREB1 | Solyc03g124110 |  |
| NAC29 | Solyc04g005610 |  |
| ERF4 | Solyc04g007000 |  |
| MADS-BOX23 | Solyc04g076700 |  |
| RAX3 | Solyc04g077260 |  |
| MYB105 | Solyc05g007870 |  |
| ERF-26 | Solyc06g035700 |  |
| MYB-SHAQKYF | Solyc07g045000 |  |
| WRKY1 | Solyc07g047960 |  |
| AGL65 | Solyc07g052700 |  |
| MYB41 | Solyc07g054840 |  |
| NAC21/22 | Solyc07g066330 |  |
| DREB1E | Solyc08g007820 |  |
| WRKY41 | Solyc08g008280 |  |
| PLATZ-TF | Solyc08g076860 |  |
| ERF5 | Solyc08g078190 |  |
| WRKY46 | Solyc08g082110 |  |
| WRKY33 | Solyc09g014990 |  |
| NAC21/22-1 | Solyc09g025310 |  |
| ERF3 | Solyc10g006130 |  |
| PsaF | Solyc02g069460 | Phostosynthesis |
| PsbQ | Solyc02g079950 |  |
| PsaH-1 | Solyc03g120640 |  |
| PsbP | Solyc06g065490 |  |
| PsaH-2 | Solyc06g066640 |  |
| PsaL | Solyc06g082940 |  |
| PsaL | Solyc06g082950 |  |
| known | Solyc06g083680 |  |
| PsbP | Solyc07g044860 |  |
| PsaG | Solyc07g066150 |  |
| PsaK | Solyc08g006930 |  |
| PsaN | Solyc08g013670 |  |
| NDH | Solyc10g006530 |  |
| psaA | Solyc10g017890 |  |
| E3-U-box-ligase | Solyc01g005160 | Protein ubiquitination |
| PUB23 | Solyc01g007010 |  |
| PUB23 | Solyc01g007030 |  |
| PUB23 | Solyc01g007040 |  |
| RNF170 | Solyc01g103970 |  |
| E3-U-box-ligase | Solyc03g114160 |  |
| RNF217 | Solyc03g115920 |  |
| E3-U-box-ligase | Solyc04g008100 |  |
| E3-U-box-ligase | Solyc04g071030 |  |
| UBC22 | Solyc06g051140 |  |
| MIEL1 | Solyc06g054540 |  |
| RBBP6 | Solyc08g061890 |  |
| AIRP2 | Solyc10g085130 |  |
| E3-Ub-Liigase-XERICO | Solyc12g006230 |  |
| ATL42 | Solyc12g007320 |  |
| Dehydrin-1 | Solyc01g109920 | Response to water |
| Dehydrin-3 | Solyc02g062390 |  |
| Dehydrin-RAB18 | Solyc02g084840 |  |
| TAS14-2 | Solyc02g084850 |  |
| 4CL | Solyc01g006640 | Peroxidase activity |
| DOX1 | Solyc02g087070 |  |
| PER22 | Solyc03g006700 |  |
| PER | Solyc03g033690 |  |
| RBOHB | Solyc05g025680 |  |
| PER52 | Solyc05g052280 |  |
| PER-1 | Solyc06g050440 |  |
| PER-2 | Solyc06g082420 |  |
| PER16 | Solyc07g017880 |  |
| PHGPx | Solyc08g080940 |  |
| PER-3 | Solyc10g076240 |  |
| PER-4 | Solyc10g084240 |  |
| NsLTP | Solyc01g105010 | Lipid binding |
| Annexin | Solyc04g073990 |  |
| CLATHRIN ASSEMBLY PROTEIN | Solyc04g074830 |  |
| MAJOR ALLERGEN PRU AR 1-LIKE | Solyc05g054380 |  |
| nsLTP | Solyc06g084190 |  |
| nsLTP | Solyc08g067500 |  |
| Hydroxycinnamoyl CoA quinate transferase 2 | Solyc08g067510 |  |
| nsLTP | Solyc09g065430 |  |
| Major allergen Mal d 1 | Solyc09g090980 |  |
| Major allergen Mal d 1 | Solyc09g090990 |  |
| nsLTP | Solyc10g075060 |  |
| nsLTP | Solyc10g075090 |  |
| nsLTP | Solyc10g075100 |  |
| no data | Solyc10g075103 |  |
| no data | Solyc10g075107 |  |
| nsLTP | Solyc10g075110 |  |
| nsLTP | Solyc10g075150 |  |
| MLP-LIKE PROTEIN 423 | Solyc05g005865 |  |
| SYNAPTOTAGMIN-3-LIKE ISOFORM X1 | Solyc09g007860 |  |
| nsLTP | Solyc10g075070 |  |
| nsLTP | Solyc06g005100 |  |
| nsLTP | Solyc09g065430 |  |
| AMD | Solyc01g010050 | Carboxy-lyase activity |
| ACS | Solyc01g095080 |  |
| TPS31 | Solyc01g101180 |  |
| TPS21 | Solyc01g101190 |  |
| CA | Solyc02g067750 |  |
| AMD | Solyc02g089610 |  |
| GD | Solyc03g098240 |  |
| B-elim.lyase dom | Solyc04g009960 |  |
| GD | Solyc04g025530 |  |
| HTD2 | Solyc06g075000 |  |
| PEPC | Solyc07g062530 |  |
| HDC | Solyc08g006740 |  |
| HDC | Solyc08g006750 |  |
| HDC | Solyc08g068600 |  |
| HDC | Solyc08g068610 |  |
| HDC | Solyc08g068630 |  |
| AADC | Solyc08g068680 |  |
| SDS | Solyc09g008670 |  |
| TS | Solyc10g005320 |  |
| ADC | Solyc10g054440 |  |
| PDC | Solyc10g076510 |  |
| ACO | Solyc12g005860 |  |
| KTI-4 | Solyc03g020010 | Peptidase inhibitor activity |
| KTI-1 | Solyc03g098670 |  |
| CPI-1 | Solyc03g098700 |  |
| CPI | Solyc03g098710 |  |
| KTI-2 | Solyc03g098720 |  |
| KTI-3 | Solyc03g098760 |  |
| KTI-4 | Solyc03g098780 |  |
| SPI | Solyc03g098790 |  |
| PI-3 | Solyc03g098795 |  |
| PI-4 | Solyc06g073587 |  |
| PI-5 | Solyc06g150133 |  |
| PI-6 | Solyc09g083435 |  |
| PI | Solyc09g084470 |  |
| PI-1 | Solyc09g089500 |  |
| PI-2 | Solyc09g089520 |  |
| SPI-1 | Solyc09g089540 |  |
| SPI-2 | Solyc11g021020 |  |
| FE2OG | Solyc01g058250 | Oxoacid metabolic process |
| ACCsynthase | Solyc01g095080 |  |
| GATase | Solyc01g106060 |  |
| Cytochrome P450 | Solyc02g092860 |  |
| MES2 | Solyc03g070380 |  |
| GD | Solyc03g098240 |  |
| GD | Solyc04g025530 |  |
| SDH | Solyc04g055030 |  |
| E1-DH | Solyc04g063350 |  |
| AS | Solyc06g007180 |  |
| P5CS | Solyc06g019170 |  |
| BCKDHA | Solyc06g059850 |  |
| HTD2 | Solyc06g075000 |  |
| LKR | Solyc07g017610 |  |
| BCAA | Solyc07g021630 |  |
| PEP carboxylase | Solyc07g062530 |  |
| THT4 | Solyc08g006730 |  |
| HDC | Solyc08g006740 |  |
| NCED5 | Solyc08g016720 |  |
| TD | Solyc09g008670 |  |
| TRPS | Solyc10g005320 |  |
| KCS1 | Solyc10g009240 |  |
| Lysine-tRNA-Ligase | Solyc10g076600 |  |
| ALD1 | Solyc11g044840 |  |
| FE2OG | Solyc11g072310 |  |
| ACO2 | Solyc12g005860 |  |
| CDKI | Solyc01g108610 | mitotic cell cycle |
| CYCLIN-D3-2 | Solyc02g092980 |  |
| CYCLIN-B2-1 | Solyc03g032190 |  |
| CDKI | Solyc03g044480 |  |
| CYCLIN-B | Solyc04g078310 |  |
| CYCLIN-B2-4 | Solyc04g082430 |  |
| SCAB2 | Solyc10g078340 |  |
| CNTD | Solyc10g080950 |  |
| CYCLIN-A1-1 | Solyc11g005090 |  |
| CNTD | Solyc12g088650 |  |
| KIN-10C | Solyc03g025470 | Microtubule motor activity |
| KIN-141 | Solyc09g075480 |  |
| KIN-14R | Solyc11g010920 |  |
| KIN-1A | Solyc01g108670 |  |
| KIN-4C-1 | Solyc04g076310 |  |
| KIN-4C-2 | Solyc06g009780 |  |
| KIN-5D | Solyc11g072820 |  |
| KIN-7A | Solyc03g119220 |  |
| KIN-Like-1 | Solyc02g084390 |  |
| KIN-Like-2 | Solyc03g114380 |  |
| KIN-Like-3 | Solyc06g075580 |  |
| KIN-Like-4 | Solyc07g065210 |  |
| KIN-Like-5 | Solyc09g090320 |  |
| KIN-Like-6 | Solyc10g083310 |  |
| KIN-Like-7 | Solyc12g005250 |  |
| KLP-3 | Solyc07g065880 |  |
| GD-1 | Solyc04g025530 | Pyridoxal phosphate binding |
| ALT | Solyc06g063090 |  |
| HDC-6 | Solyc08g068600 |  |
| HDC-1 | Solyc08g068610 |  |
| HDC-2 | Solyc08g068630 |  |
| HDC-3 | Solyc08g068680 |  |
| TDH | Solyc09g008670 |  |
| ALD1 | Solyc11g044840 |  |
| OAT | Solyc08g048450 |  |
| TAT | Solyc10g007110 |  |
| AGXT | Solyc10g076250 |  |
| HDC-4 | Solyc08g006750 |  |
| GD-2 | Solyc03g098240 |  |
| HDC-5 | Solyc08g006740 |  |
| CTH | Solyc08g083110 |  |
| AP | Solyc05g016310 | Peptidase activity |
| pcs1-like | Solyc07g045100 |  |
| CYP-3 | Solyc02g080880 |  |
| 4-mmp | Solyc11g011440 |  |
| APF-2 | Solyc07g041900 |  |
| SAG39 | Solyc10g018750 |  |
| CTP | Solyc07g045100 |  |
| CP14 | Solyc02g076910 |  |
| Ftsh 6 | Solyc03g059260 |  |
| Sag12 | Solyc02g076710 |  |
| Sbt1.5 | Solyc02g081550 |  |
| CBP1 | Solyc02g077040 |  |
| pppde-1 | Solyc02g021220 |  |
| GGP-5 | Solyc04g077650 |  |
| LON peptidase | Solyc02g082060 |  |
| PREPL | Solyc07g006090 |  |
| Egy3 | Solyc10g009020 |  |
